# Development of the gut microbiota in healthy twins during the first 2 years of life and associations with body mass index z-score: Results from the Wuhan twin birth cohort study

**DOI:** 10.3389/fmicb.2022.891679

**Published:** 2022-08-18

**Authors:** Hong Mei, Shaoping Yang, An’na Peng, Ruizhen Li, Feiyan Xiang, Hao Zheng, Yafei Tan, Ya Zhang, Ai’fen Zhou, Jianduan Zhang, Han Xiao

**Affiliations:** ^1^Wuhan Children’s Hospital (Wuhan Maternal and Child Healthcare Hospital), Tongji Medical College, Huazhong University of Science and Technology, Wuhan, China; ^2^Department of Maternal and Child Health Care, School of Public Health, Tongji Medical College, Huazhong University of Science and Technology, Wuhan, China

**Keywords:** gut microbiota, twins, body mass index z-score, birth cohort study, group-based trajectory model, linear mixed-effect model

## Abstract

The gut microbiota undergoes rapid and vital changes to microbial community structure and the microbial-immune crosstalk during the first 3 years of life, which is thought to be involved in the pathobiology of later-life disease. Compared to single-born children, little is known about the gut microbiota of twins in early childhood. Based on the Wuhan Twin Birth Cohort study, 344 stool samples from 204 twin families were analyzed to investigate the difference in gut microbiota composition at 6, 12, and 24 months of age. Furthermore, this study evaluated the association between gut microbiota development curves and body mass index z-score (BMI_Z) curves at 6, 12, and 24 months of age. The predominant microbiota phyla identified in twins were *Proteobacteria, Actinobacteriota, Firmicutes, Bacteroidota*, and *Verrucomicrobiota*. The richness and diversity of gut microbiota increased from 6 to 24 months old (alpha diversity with *p* < 0.05). Beta diversity revealed 61 gut microbiota genera that were significantly different in relative abundance among the three age groups. Among the 61 gut microbiota genera, 30 distinct trajectory curves (DTCs) were generated by group-based trajectory models after log2 transformation of their relative abundance. Subsequently, Spearman correlation analysis revealed that only five gut microbiota DTC were correlated with the BMI_Z DTC. Therefore, we further examined the association between the five gut microbiota genera DTC and BMI_Z DTC using generalized estimation equation models. The results revealed a significant association between the DTC groups of *Parabacteroides* and that of BMI_Z (coefficient = 0.75, *p* = 0.04). The results of this study validated the hypothesis that the richness and diversity of gut microbiota developed with age in twins. Moreover, participants with a higher DTC of log2-transformed *Parabacteroides* had a higher BMI_Z DTC during the first 2 years of life. Further studies are needed to confirm the association between *Parabacteroides* and BMI_Z in other populations.

## Introduction

The human gut hosts a dynamic microbial ecosystem, namely, the gut microbiota, which has an extensive metabolic repertoire and is increasingly recognized to provide essential benefits to its host. These include dietary energy extraction, development and maintenance of the immune system, and protection against pathogens (Tun et al., 2018; Valdes et al., 2018; Parker et al., 2020). During the first 2–3 years of life, the gut microbiota undergoes rapid and vital changes to microbial community structure. The microbial-immune crosstalk during this period is thought to be involved in the pathobiology of later-life disease (Virtanen et al., 2014; Stewart et al., 2017; Stewart et al., 2018; Kitamoto et al., 2020; Chen et al., 2021).

The gut microbiota is easily influenced by environmental factors, such as delivery mode, feeding pattern, antibiotic use, etc., especially during the early years of life, leading to differences in gut microbiota composition in different populations (Yasmin et al., 2017; Korpela et al., 2020; Li et al., 2021). A comprehensive review of cross-sectional studies performed in 2020 reported that the most prevalent bacteria in meconium samples were *Staphylococcus*, followed by *Enterobacteriaceae, Enterococcus, Lactobacillus*, and *Bifidobacterium*. These are gradually replaced by obligate anaerobic bacteria, such as the Firmicutes, *Clostridiaceae*, and *Lachnospiraceae* (Mesa et al., 2020). In contrast, another cohort study using fecal samples of infants aged 1, 6, 12, and 24 months found that the abundance of *Bacteroides, Lachnospiraceae* unclassified, *Faecalibacterium, Akkermansia*, and *Phascolarctobacterium* rapidly increased after 6 months, while *Escherichia, Bifidobacterium*, Veillonella, and *Streptococcus* decreased in abundance over time (Sugino et al., 2021).

Compared to single-born neonates, twins have higher risks of preterm birth, low birth weight, C-section delivery at birth, diseases during infancy, and a higher catch-up weight gain (Ruixiang et al., 2021; Duy Anh et al., 2022; Hessami et al., 2022; Zhao et al., 2022). All these factors may influence the development of gut microbiota. Currently, there is a lack of studies focusing on the composition of gut microbiota in twin infants. The only longitudinal study on twins’ gut microbial development was published in 2021. It reported the development of gut microbiota each week and distinguished the difference in gut microbiome diversity and composition between males and females in preterm twins (Chen et al., 2021). However, this study focused on preterm twins with gut microbiota detected during the first 28 days of life, and it did not exclude preterm twins with antibiotics exposure, which may limit the generalizability of the results to other twin populations.

It is widely accepted that there is a significant association between gut microbiota and obesity (Vallianou et al., 2019; Gupta et al., 2020; Frank et al., 2021). However, the negative association between the gut microbiota and obesity/BMI/BMI_Z/weight during the early years of life remains unproven. The diverging conclusions may stem from the dynamic balance of gut microbiota and differences in study populations and environmental factors.

Based on the Wuhan Twin Birth Cohort study (WTBC) (Zhao et al., 2017; Yang et al., 2018), 344 stool samples without antibiotics exposure within the first year of life were collected from 6 months old to 24 months old. The samples were analyzed to investigate the difference in gut microbiota development at different ages and evaluate the association between the gut microbiota and BMI_Z.

## Materials and methods

### Participants included

Twins were generated from the WTBC study born between October 2016 and December 2020, conducted in Wuhan Children’s Hospital (Wuhan Maternal and Child Healthcare Hospital), Tongji Medical College, Huazhong University of Science and Technology, Wuhan, China. The including criteria of twins in the study were: (1) born at the study hospital without congenital disease; (2) had at least two follow-ups with stool samples or anthropometric data collected at 6, 12, and 24 months of age; (3) no antibiotic used during the first year of life. This study was approved by the Ethics Committee of Wuhan Children’s Hospital (Wuhan Maternal and Child Healthcare Hospital) (IRB number: WHFE2016050).

### Data collection

Face-to-face interviews were conducted within 30 days of the twins reaching 6 months of age and within 90 days of the twins reaching 12 and 24 months of age. At each follow-up, fresh stool samples were collected in a sterile plastic container 1 or 2 days before the interview by caretakers and were immediately frozen at –20°C at home and transferred into the –80°C freezer within 1 h after arriving at the study hospital. During the interview, the twins’ weight and height were measured by trained nurses, with weight measured to the nearest 50 g and height measured to the nearest 0.1 cm. Both weight and height were measured in duplicate, and the mean of the values was used in the analyses. Information about the maternal educational level, delivery age, pre-pregnant BMI, delivery mode, gestational age at delivery, twins’ sex, birth weight and height, feeding pattern at 1 and 6 months old, and antibiotics intake during the first year of life was recorded.

### Variables generation

Body mass index was calculated as weight (kg) divided by the square of the height (m^2^), and BMI z-scores were calculated by using the age- and sex-specific BMI reference from WHO Child Growth Standards (2006) (World Health Organization, 2006). The maternal educational level was classified as middle school or under, high school/technical, university/college, or advanced. The delivery mode was divided into vaginal birth or C-section delivery, and the sex was categorized as male or female. Feeding patterns at 1, 6, and 12 months of age were categorized as exclusive breastfeeding, mixed feeding, and formula feeding. Maternal delivery age was the difference between the date of birth of the twins and the mother. Pre-pregnant BMI was calculated as pre-pregnant weight divided by the square of the height. Gestational age at delivery was calculated as the difference between the twins’ birth date and maternal last menstrual date. The zygosity of the twins was also detected using neonatal blood spot samples by short tandem repeats genetic typing. They were categorized as monozygotic or dizygotic twins.

### High-throughput sequencing of 16S rRNA gene

The QIAamp PowerFecal DNA Kit (QIAGEN, Germantown, MD, United States) was used to extract DNA from stool samples, and pooled DNA ≥ 200 μg was used for Illumina sequencing. The primer pair 341F (CCTAYGGGRBGCASCAG) and 806R (GGACTACHVGGGTWTCTAAT) were used to amplify the V3–V4 region of the 16S rRNA gene. TruSeq DNA PCR-Free Sample Preparation Kit (Illumina, United States) was adopted to generate the sequencing libraries, and the library quantity was determined by Qubit 2.0 Fluorometer (Thermo Fisher Scientific, MA, United States). Then, the libraries were sent for sequencing by Illumina NovaSeq6000 (Illumina, United States) at Novogene Co. Ltd (Beijing China). The raw data generated from the Illumina NovaSeq6000 platform were paired-end reads. To merge reads of the same DNA fragment, FLASH (Version 1.2.7) was used to identify the splicing sequences (Magoč and Salzberg, 2011). Based on the unique barcode, the barcode and primer sequence were removed with QIIME (version 1.9.1^1^) (Caporaso et al., 2010). To control the sequencing quality, raw tags with low quality (quality value ≤ 19, homopolymers ≥ 3 bases, and sequence length ≤ 370 bp) were filtered by QIIME. Tags were compared with VSEARCH (version 2.17.1) to remove chimera sequences, and the effective reads were finally obtained (Rognes et al., 2016). Sequence analysis was performed by Uparse software (Uparse v7.0.1001^2^) (Edgar, 2013). Sequences with more than 97% similarity were assigned as the same Operational Taxonomic Units (OTUs). The representative sequence for each OTU was screened for further annotation. For each representative sequence, the Silva138 database^3^ was used based on the MUSCLE classifier (Version 3.8.31^4^) algorithm to annotate taxonomic information (Quast et al., 2013).

### Statistical analysis

For epidemiological data, the descriptive characteristics of the participants were expressed as mean (SD) for normally distributed variables, and as frequency (percentage) for categorical variables. Variance analysis (ANOVA) was used to determine the difference of normally distributed variables among the three age groups, and the chi-square test was used to evaluate the significance of categorical variables.

To study the phylogenetic relationship of different OTUs and the predominant species in different age groups, a Venn plot was used to identify the difference in OTUs, and a bar plot was used to illustrate the top 10 or 20 microbiota in phylum or genus taxa, respectively. Furthermore, the changes in predominant microbiota among each age group were illustrated in a Sankey diagram, which was conducted using the MUSCLE software (version 3.8.31, see text footnote 4). Hierarchical clustering of the 35 most abundant genera that were significantly different among the three age groups in relative abundance (*p* < 0.05; ANOVA) was visualized using a heatmap.

Alpha diversity was measured using the Chao1 index (which measures the overall richness of a community) and Shannon diversity index (which measures the overall diversity of a community, including the number of taxa/OTUs) with QIIME (version 1.7.0). As non-normal distribution data, the Chao1 index was log2-transformed to obtain a normal distribution. Linear mixed-effect (LME) models were used to make inferences for the age-specific longitudinal alpha-diversity data in SAS 9.4. In LME models, the twins’ family was set as the random effect, while the twins’ zygosity, sex, birth weight, gestational age, feeding pattern at 1 month old, maternal delivery age, and educational level were the controlling variables.

Beta-diversity analysis was measured based on Bray–Curtis distance matrices for individuals sampled at each age group using the Kruskal–Wallis test. Principal coordinates analysis (PCoA) was performed to discriminate principal coordinates and visualize signatures among the three age groups. Similarity percentage analysis (SIMPER analysis) was adopted to further identify the gut microbiota at the genus level that showed significant differences in relative abundance among the three age groups with a contribution rate for difference > 0.001 with simple function in vegan package, R software. Linear discriminant analysis Effect Size (LEfSe) was used with R software to identify the predominant microbiota genera at different ages, considering biological consistency and effect relevance. Significance was accepted as *p* < 0.05 following false discovery rate (FDR) correction.

Based on the results from SIMPER and LEfSe analyses, gut microbiota genera that were significantly different among the three age groups were included in the association analysis with the twins’ body mass index z-score. Subsequently, the relative abundance of each candidate microbiota genera at each time point were log2-transformed to obtain a normal distribution. Then, group-based trajectory models (GBTMs) (Nagin and Land, 1993) were used to evaluate the development of log2-transformed microbiota genera and BMI_Z by SAS 9.4 TRAJ package. GBTM assumes that all participants are from the same population but composed of distinct groups defined by their development trajectory, say distinct trajectory curve (DTC). Model selection is a key issue in GBTM, and we followed suggestions from early studies to complete the task (Nagin, 2014; Lo-Ciganic et al., 2022). Details of model selection and SAS script for GBMT were detailed in Supplementary Appendix 1. Based on the GBTM, the trajectory of log-transformed microbiota genera and BMI_Z could be divided into different groups. In this study, we successfully generated two different trajectory curves for both log-transformed microbiota genera and BMI_Z in the end. Thus, we defined these with a stable trend far from the null score as high DTC group and these with a stable trend near the null score as low DTC group for both log-transformed microbiota genera and BMI_Z. Then, Spearman correlation was used to determine the relationship between GBTM-categorized DTC of relative abundance of gut microbiota genera and BMI_Z to identify microbiota genera that were correlated with gut microbiota genera for further association analyses. Thus, generalized estimation equation models (GEE, PROC GENMOD in SAS 9.4) were applied to examine the association between DTC groups of log2-transformed relative abundance of gut microbiota genera and BMI_Z while adjusting for the twins’ family, zygosity, sex, birth weight, gestational age, feeding pattern at 1 month old, maternal delivery age, and educational level (SAS script was detailed in Supplementary Appendix 2).

The statistical significance was defaulted as *p* < 0.05 in our analyses.

## Results

### Basic characteristics of twins

A total of 344 stool samples were collected from 204 twins in 129 families (75 families with both twins and 54 families with only one twin included), among which 149, 130, and 65 stool samples were collected at 6, 12, and 24 months of age, respectively. The basic characteristics are summarized in Supplementary Table 1. Furthermore, the age-specific characteristics were calculated to account for data missing among different age groups; no significant difference was found for the variables listed in Supplementary Table 1 among the three age groups.

### Community of the gut microbiota

**FIGURE 1:**
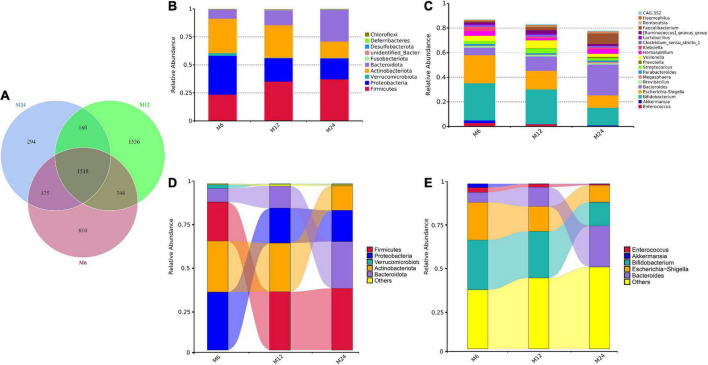
Community of the gut microbiota among the three age groups. M6: 6 months old group; M12: 12 months old group; M24: 24 months old group. **(A)** Venn plot illustrating the number of shared and unique OTUs in the three age groups. **(B)** The 10 most abundant gut microbiota phyla in the three age groups. **(C)** The 20 most abundant gut microbiota genera in the three age groups. **(D)** The Sankey diagram demonstrating the changes of the five main microbiota phyla from 6 to 24 months old. **(E)** The Sankey diagram demonstrating the changes of the five main microbiota genera from 6 to 24 months old.

A total of 36.5 million quality-filtered reads with an average of 102,586 reads per sample were detected after sequencing. In total, 5649 OTUs were identified, among which 1,518 were shared by the three age groups (Figure 1A). The OTUs were then classified into 55 phyla, 136 classes, 330 orders, 497 families, and 894 genera in total (Supplementary Table 2 and Supplementary Taxa). *Firmicutes, Proteobacteria, Actinobacteriota*, and *Bacteroidota* were the most abundant phyla in relative abundance, while *Bifidobacterium, Escherichia-Shigella, Bacteroides*, and *Veillonella* were among the most abundant genera in relative abundance (Figures 1B,C). The Sankey diagram illustrates the changes of five main microbiota from 6 to 24 months old. As displayed in Figures 1D,E, a significant increase of *Firmicutes* phyla in relative abundance was observed from 6 to 12 months old, while the relative abundance of *Bacteroidota* phyla and *Bacteroides* genera increased from 12 to 24 months. A marked decrease in relative abundance of *Proteobacteria* phyla and *Enterococcus* genera was observed from 6 to 12 months old, and a decrease in relative abundance occurred in the *Actinobacteriota* phyla and *Bifidobacterium* genera and *Escherichia-Shigella* genera from 12 to 24 months old. Furthermore, a heatmap was used to detail the 35 most common bacterial among the three age groups at the phylum and genus levels (Supplementary Figure 1).

### The alpha diversity of gut microbiota among twins

**FIGURE 2:**
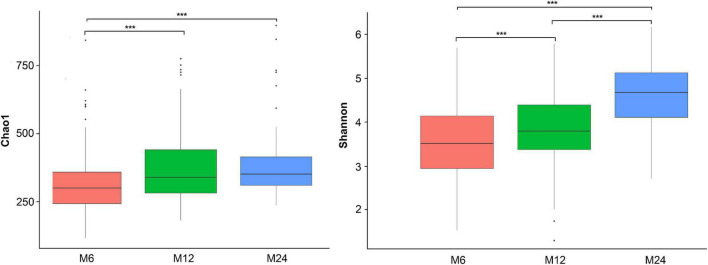
Alpha diversity of the gut microbiota. M6: 6 months old group; M12: 12 months old group; M24: 24 months old group. ***Indicated significant difference between groups with *p* < 0.0001. The left one is the Chao1 index, and the right one is the Shannon index.

The alpha-diversity analysis showed that the Chao1 index (richness measure) and the Shannon diversity index (diversity measure) increased from 6 to 24 months old (Figure 2). Considering some missing samples, the diversity among the three age groups was analyzed using linear mixed-effect models (LME) while adjusting for the twins’ zygosity, sex, birth weight, gestational age, feeding pattern at 1 month old, and maternal delivery age, educational level and twin family as random effect. The LME results confirmed the increasing trend of both Chao1 and Shannon diversity indices with age (Supplementary Table 3).

### Differential analysis of beta diversity among the three age groups

**FIGURE 3:**
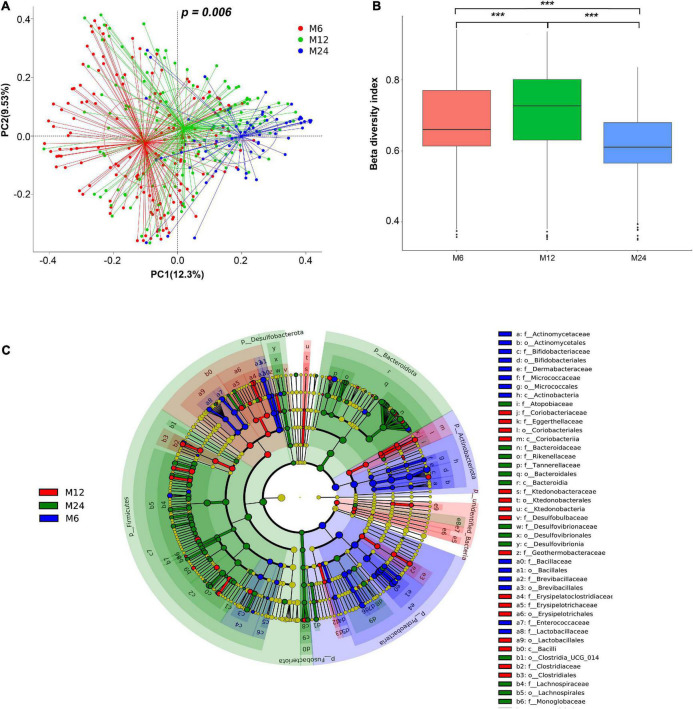
Beta diversity of the gut microbiota. M6: 6 months old group; M12: 12 months old group; M24: 24 months old group. ***Indicated significant difference between groups with *p* < 0.0001. **(A)** Principal coordinates analysis (PCoA) of twins at the three age groups. **(B)** Difference of beta-diversity index among the three age groups. Beta-diversity index refers to the inter-individual Bray–Curtis dissimilarity. **(C)** Cladogram showing gut microbiota that was significantly different among the three age groups. f_: family level; o_: order level; and c_: class level.

The PCoA plot revealed that bacterial signatures among the three age groups were significantly distinct (Figure 3A, *p* = 0.006). Results from the Kruskal–Wallis test showed significant differences in beta-diversity dissimilarity indices among the three age groups (Figure 3B).

In total, 61 microbiota genera were significantly different among the three age groups, with a contribution rate for difference > 0.001. The 61 microbiota genera are listed in Supplementary Table 4. LEfSe was used to further compare the compositional differences of gut microbiota among the three age groups at all taxa. As illustrated in Figure 3C, the relative abundance of 14 microbiota classes, 16 microbiota orders, and 13 microbiota families was significantly different among the three age groups (Figure 3C). The 61 gut microbiota genera listed in Supplementary Table 4 were obtained based on the candidate microbiota identified in the SIMPER and LEfSe analyses.

### Association between distinct trajectory curve of log-transformed relative abundance of gut microbiota genera and body mass index z-score

Among the 61 log2-transformed gut microbiota genera, 30 microbiota genera were successfully categorized into two DTC groups, namely, high DTC group and low DTC group. BMI_Z was also categorized into high DTC group and low DTC group. Then, Spearman correlation analysis revealed a significant correlation between the DTC groups of five log2-transformed microbiota genera, *Akkermansia, Parabacteroides, Clostridium innocuum group, Roseburia*, and *Anaerostipes*, and those of BMI_Z (Supplementary Table 5), respectively. Then, GEE models were used to assess the association between the DTC groups of the five log2-transformed microbiota genera and those of BMI_Z, revealing that the DTC groups of both log2-transformed *Parabacteroides* and *Anaerostipes* were associated with BMI_Z DTC groups with coefficients 0.70 and –0.64, respectively (*p* < 0.05 with no variables controlled for, Table 1). After controlling for the twins’ zygosity, sex, birth weight, gestational age, feeding pattern at 1 month old, maternal delivery age, and educational level, a significant association was only found between log2-transformed *Parabacteroides* DTC groups and BMI_Z DTC groups (coefficient = 0.75, *p* = 0.04, Table 1).

**TABLE 1:** Association between distinct trajectory curve groups of gut microbiota and BMI_Z: results from generalized estimation equation models.

	Unadjusted model			Adjusted model		
	Coefficient	95% CI	*p*	Coefficient	95% CI	*p*
*Akkermansia*	0.89	–0.01, 1.80	0.05	0.85	–0.17, 1.87	0.10
*Parabacteroides*	0.70	0.01, 1.39	0.04	0.75	0.02, 1.49	0.04
*Clostridium innocuum group*	–0.57	–1.15, 0.01	0.05	–0.57	–1.21, 0.06	0.07
*Roseburia*	0.57	–0.01, 1.15	0.05	0.57	–0.06, 1.21	0.08
*Anaerostipes*	–0.64	–1.22, –0.07	0.03	–0.47	–1.11, 0.17	0.15

In the adjusted models, twins’ zygosity, sex, birth weight, gestational age, feeding pattern at 1 month old, maternal delivery age, and educational level were controlled for.

## Discussion

To our knowledge, this is the first study to examine the difference of gut microbiota diversity in twins at different periods and to evaluate the correlation between gut microbiota development and BMI_Z in twins. There was a significant increase in alpha diversity (both richness and diversity) with age in the studied twins. Significant differences in beta diversity were also detected among the three age groups, as expected. In association analyses, an association between the high log2-transformed *Parabacteroides* DTC was found and the high BMI_Z DTC group.

The establishment of stable gut microbiota in singletons generally usually relies on two major transitions in infancy. The first transition occurs soon after birth, during lactation, and results in the dominance of *Bifidobacterium* (Backhed et al., 2015). Then, during the weaning period, with the introduction of solid foods, the second transition occurs to yield an adult-type complex gut microbiota dominated by the phyla *Bacteroidetes* and *Firmicutes* (Bergstrom et al., 2014). At around 3 years of age, gut microbiota is dominated by *Bacteroides, Prevotella*, and other *Firmicutes*, which is similar to adults (Fouhy et al., 2019; Zhu et al., 2021). In this study, similar patterns were identified in the development of the gut microbiota in twins. The most abundant gut microbiota at 6 months old was *Bifidobacterium* and was *Bacteroides* at 24 months old. In contrast, *Firmicutes* and *Bacteroidetes* increased after 6 months of age (Figure 1). Apart from the most abundant gut microbiota, the 10 most common phyla and 20 most common genera of gut microbiota in the three age groups were illustrated, respectively, representing similar findings in singletons (Backhed et al., 2015; Olsson et al., 2022; Vacca et al., 2022). Considering the difference between twins and singletons at birth, the similarity of predominant gut microbiota between twins and singletons may indicate that the influence of C-section delivery, preterm birth, and low birth weight may disappear after 6 months of age, as reported by Fiona et al. Previous studies also reported that there were no significant differences at the phylum level between the preterm and full-term children or delivery modes at one and 2 years old (Yasmin et al., 2017; de la Cuesta-Zuluaga et al., 2019; Fouhy et al., 2019; Chen et al., 2021). More studies are needed to confirm if gut microbiota was similar between twin and singleton children.

Previous studies in singletons reported a significant difference in gut microbiota diversity in the first 3 years of life (Yatsunenko et al., 2012; Vu et al., 2021). In this study, the overall richness of communities was observed lower at 6 months of age compared to 12 and 24 months, but similar between 12 and 24 months. Meanwhile, the diversity increased from 6 months of age to 24 months as beta-diversity analysis revealed a significant difference among the three age groups, confirmed by the PCoA plot. Considering the confounding variables for the twins’ gut microbiota, LME models were used to confirm the results while adjusting for twins’ zygosity, sex, birth weight, gestational age, feeding pattern at 1 month old, maternal delivery age, and educational level. Numerous studies have investigated the difference in gut microbiota among children of different ages, but few studies have been carried out on twins. A direct comparison between our study and others is not possible, given the differences in the study population. However, most of the specific gut microbiota we distinguished among the 6-, 12- and 24-months’ old groups were similar to those reported in the study by Kameron et al. in singletons of the same age groups (Sugino et al., 2021).

Longitudinal studies on gut microbiota and BMI were rare, especially in early childhood, due to the difficulty of stool samples collection and the data analysis of gut microbiota. This study analyzed the gut microbiota of 204 twins from 129 families at three age stages and demonstrated that five gut microbiota, genera *Akkermansia, Parabacteroides, Clostridium innocuum group, Roseburia*, and *Anaerostipes*, were correlated with BMI_Z. As short-chain fatty acid producers, *Akkermansia, Roseburia*, and *Anaerostipes* were previously reported to be associated with obesity in animal models and adults (Pinart et al., 2021; Gao et al., 2022; Pai et al., 2022; Xu et al., 2022; Zou et al., 2022). There were only two studies that reported a positive association between *Akkermansia* and BMI_Z in early childhood in singleton children, which was consistent with our findings (Houtman et al., 2022; Reyna et al., 2022). *Clostridium innocuum group* is one of the probiotics that has prebiotic effects on gut dysbiosis (Bian et al., 2022). In this study, Spearman correlation analysis found a significant correlation between DTC of log2-transformed *Clostridium innocuum group* and BMI_Z. We infer that *Clostridium innocuum group* may have a protective effect against high BMI_Z, which is consistent with results from a cohort study on the association between the abundance of Clostridium at 1 year old and BMI_Z growth curves from birth to 5 years old (Reyna et al., 2022). After adjusting for confounding variables, *Parabacteroides* were found to be positively associated with BMI_Z in this study. In previous studies, *Parabacteroides* were usually reported to be significantly increased with a low-carbohydrate diet-induced weight loss in mice models and adults (Wang et al., 2022; Yoshida et al., 2022; Yu et al., 2022). In this study, although we did not consider the influence of diet, we detected the significant associations between *Parabacteroides* and BMI_Z, which indicated that the alterations might not be specific to the low-carbohydrate diet reported by Fragiadakis et al. (2020).

Our study adds to the literature on the development of gut microbiota and its associations with BMI_Z. To the best of our knowledge, this is the first investigation to be reported in twins at an early stage of life with both gut microbiota and BMI_Z at three critical time points of twins’ growth considered, which enabled us to assess the associations between gut microbiota and BMI_Z in time sequence. It is known that systemic antibiotic administration in early infancy has both short- and long-term consequences on the gut microbiome (Yallapragada et al., 2015; Kwon et al., 2022). This study excluded twins who were exposed to antibiotics during infancy. Moreover, for comparison or association analyses, potential factors like sex, delivery mode, gestational age, and basic maternal characteristics were adjusted for, as they might influence both gut microbiota and BMI_Z (Yasmin et al., 2017; Chen et al., 2021; Zhao et al., 2022). However, the following limitations should be considered when interpreting our findings. There is a lack of gut microbiota data for twin pregnancies during pregnancy and for twins less than 6 months old, which might influence children’s gut microbiota in later life (Fouhy et al., 2019; Korpela et al., 2020; Sugino et al., 2021). Although we included many confounders in the current study, we did not detail the feeding patterns for infants after 6 months old when the increased food variety may influence the composition of gut microbiota (Tang et al., 2021). Another limitation is the relatively small sample size, which might reduce the statistical power. In the GEE models, we displayed the results without multiple testing, when the *p* values were adjusted for, and the *p*-values were more than 0.05 for all the five microbiota genus after co-variables controlled for Supplementary Table 6. Thus, it is hard to determine whether the null association between gut microbiota and BMI_Z was only a false negative. Furthermore, the sample size of monozygotic twins was small with three time points’ stool sample analyzed, and in the LME models, as random effects, zygosity did not have a significant influence on BMI_Z; thus, we did not analyze the effect of monozygotic and dizygotic twins separately.

## Conclusion

This is the first study to provide preliminary evidence that early-life gut microbiota development may be associated with BMI_Z before 2 years of life in twins. Notably, this study reported for the first time the domain gut microbiota in twins at 6, 12, and 24 months of age without antibiotic exposure. Additionally, the association between gut microbiota and BMI_Z was conducted by evaluating the association between DTC of the log2-transformed gut microbiota and BMI_Z, which made it easy to understand the relationship between gut microbiota and BMI_Z in time sequence. However, further studies with a large population are required to corroborate these findings.

## Data availability statement

The raw sequence data reported in this manuscript have been deposited in the Genome Sequence Archive (Genomics, Proteomics and Bioinformatics 2021) in National Genomics Data Center (Nucleic Acids Research 2022), the China National Center for Bioinformation/Beijing Institute of Genomics, Chinese Academy of Sciences (GSA: CRA007379) that are publicly accessible at https://ngdc.cncb.ac.cn/gsa.

## Ethics statement

This study was approved by the Ethics Committee of Wuhan Children’s Hospital (Wuhan Maternal and Child healthcare Hospital) (IRB number: WHFE2016050). Written informed consent to participate in this study was provided by the participants’ parents.

## Author contributions

HM, RL, FX, HZ, AP, and YT coordinated the Wuhan Twin Birth Cohort study and collected the data. HM, SY, RL, AZ, and HX designed the study and obtained funding. HM and YZ analyzed the data. HM, YZ, JZ, AZ, and HX interpreted the results and wrote and edited the manuscript. All authors critically reviewed the manuscript and approved the final version for submission.

## Footnotes

1
http://qiime.org/scripts/split_libraries_fastq.html

2
http://drive5.com/uparse/

3
http://www.arb-silva.de/

4
http://www.drive5.com/muscle/
