# Supplementary figures and images for: Development of the gut microbiota in healthy twins during the first 2 years of life and associations with body mass index z-score: Results from the Wuhan twin birth cohort study
Source: Front Microbiol. 2022 Aug 18;13:891679. doi: 10.3389/fmicb.2022.891679 (PMC9433903; doi:10.3389/fmicb.2022.891679)

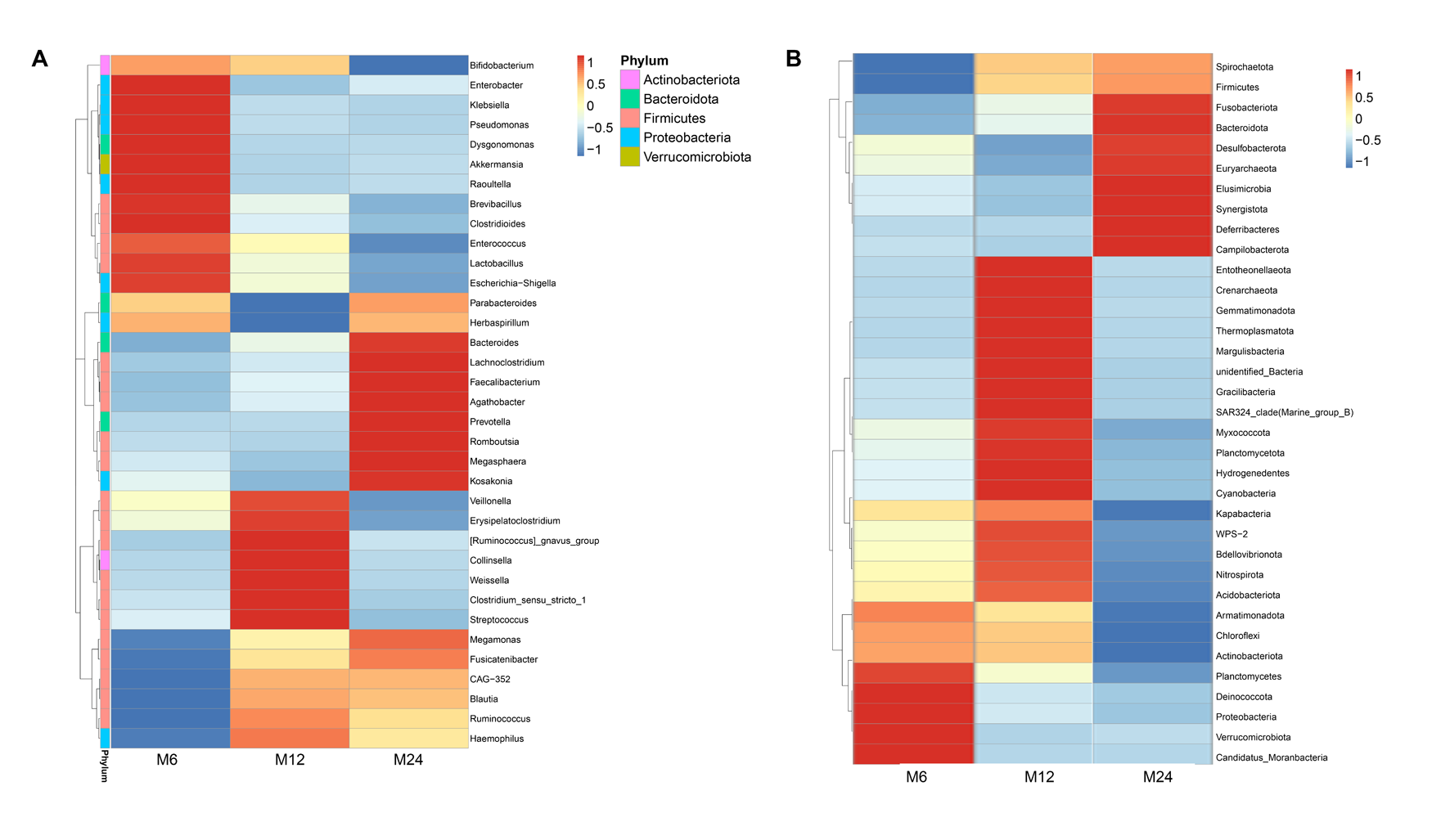

Supplement: Supplementary file 1 [file Image_1.tif]
