# Supplementary material for: Development of the gut microbiota in healthy twins during the first 2 years of life and associations with body mass index z-score: Results from the Wuhan twin birth cohort study
Source: Front Microbiol. 2022 Aug 18;13:891679. doi: 10.3389/fmicb.2022.891679 (PMC9433903; doi:10.3389/fmicb.2022.891679)
Supplement: Supplementary file 2 [file Data_Sheet_1.docx]

Supplementary table 1. Basic characteristics of the participants [means (SD)/ n (%)]

|  | Total (N=204) | Data missing | 6 month-old group | 12 month-old group | 24 month-old group |
| --- | --- | --- | --- | --- | --- |
| Twins’ information |  |  |  |  |  |
| Zygosity |  | 10 (4.90) |  |  |  |
| Monozygosity | 72 (37.11) |  | 49 (37.69) | 64 (37.65) | 32 (35.56) |
| Dyzigosity | 122 (62.89) |  | 81 (62.31) | 106 (62.35) | 58 (64.44) |
| Sex |  | 0 (0.00) |  |  |  |
| Boy | 107 (52.45) |  | 65 (47.79) | 94 (53.11) | 53 (53.00) |
| Girl | 97 (47.55) |  | 71 (52.21) | 83 (46.89) | 47 (47.00) |
| Gestational age (week) | 36.87 (1.18) | 2 (0.98) | 36.91 (0.94) | 37.02 (0.91) | 36.72 (1.55) |
| Preterm birth | 76 (37.25) | 2 (0.98) | 53 (38.97) | 53 (29.94) | 37 (37.00) |
| Delivery mode |  | 2 (0.98) |  |  |  |
| Vaginal birth | 4 (1.98) |  | 2 (1.47) | 4 (2.29) | 3 (3.06) |
| C-section | 198 (98.02) |  | 134 (98.53) | 171 (97.71) | 95 (96.94) |
| Birth weight (g) | 2549.46 (367.70) | 2 (0.98) | 2575.15 (357.03) | 2558.74 (348.01) | 2487.86 (378.79) |
| Birth length (cm) | 47.27 (2.09) | 2 (0.98) | 47.43 (1.89) | 47.33 (1.98) | 46.85 (2.29) |
| Feeding pattern at 1 month-old |  | 10 (4.90) |  |  |  |
| Exclusive breastfeeding | 25 (12.89) |  | 22 (16.54) | 18 (10.78) | 11 (11.70) |
| Mixed feeding | 148 (76.29) |  | 95 (71.43) | 133 (79.64) | 75 (79.79) |
| Formula feeding | 21 (10.82) |  | 16 (12.03) | 16 (9.58) | 8 (8.51) |
| Weight at 6 month- old (kg) | 7.98 (0.93) | 68 (33.33) | 7.98 (0.93) | 7.94 (0.91) | 7.85 (0.85) |
| Length at 6 month-old (cm) | 66.98 (2.42) | 68 (33.33) | 66.98 (2.42) | 67.03 (2.48) | 67.14 (2.49) |
| BMI_Z at 6 month-old | 0.39 (1.01) | 68 (33.33) | 0.39 (1.01) | 0.32 (1.00) | 0.14 (0.99) |
| Feeding pattern at 6 month-old |  | 74 (36.27) |  |  |  |
| Exclusive breastfeeding | 9 (6.92) |  | 9 (6.92) | 6 (5.50) | 2 (3.45) |
| Mixed feeding | 67 (51.54) |  | 67 (51.54) | 58 (53.21) | 35 (60.34) |
| Formula feeding | 54 (41.54) |  | 54 (41.54) | 45 (41.28) | 21 (36.21) |
| Weight at 12 month- old | 9.93 (1.27) | 27 (13.24) | 9.86 (1.32) | 9.93 (1.27) | 9.75 (1.16) |
| Length at 12 month-old | 75.62 (3.38) | 27 (13.24) | 75.50 (3.49) | 75.62 (3.38) | 75.39 (2.91) |
| BMI_Z at 12 month-old | 0.54 (1.20) | 27 (13.24) | 0.51 (1.26) | 0.54 (1.20) | 0.38 (1.08) |
| Feeding pattern at 12 month-old |  | 27 (13.24) |  |  |  |
| Mixed feeding | 36 (20.34) |  | 32 (28.57) | 36 (20.34) | 19 (20.21) |
| Formula feeding | 141 (79.66) |  | 80 (71.43) | 141 (79.66) | 75 (79.79) |
| Maternal information |  |  |  |  |  |
| Maternal age at delivery (year) | 30.32 (4.11) | 2 (0.98) | 30.52 (4.10) | 30.37 (4.12) | 30.66 (4.79) |
| Maternal educational level |  | 2 (0.98) |  |  |  |
| Middle school or less | 73 (36.14) |  | 50 (36.76) | 65 (37.14) | 36 (36.73) |
| High school | 82 (40.59) |  | 55 (40.44) | 66 (37.71) | 40 (40.82) |
| College or above | 47 (23.27) |  | 31 (22.79) | 44 (25.14) | 22 (22.45) |
| Weight at 24 month- old | 12.24 (1.400 | 104 (50.98) | 12.38 (1.42) |  | 12.24 (1.40) |
| Length at 24 month-old | 88.21 (3.51) | 104 (50.98) | 88.80 (3.40) |  | 88.21 (3.51) |
| BMI_Z at 24 month-old | -0.02 (0.88) | 104 (50.98) | -0.02 (0.99) |  | -0.02 (0.88) |

Supplementary table 2. Numbers of microbiota of each taxa in the three age groups.

| Taxa | 6 month-old group | 12 month-old group | 24 month-old group | Total |
| --- | --- | --- | --- | --- |
| Phylum | 36 | 54 | 24 | 56 |
| Class | 94 | 133 | 64 | 137 |
| Order | 232 | 325 | 144 | 334 |
| Family | 364 | 489 | 222 | 504 |
| Genus | 687 | 855 | 453 | 895 |

Supplementary table 3. Comparison of the Chao1 and Shannon indices among different the three age groups, results from a linear mixed effect model.

|  | The Chao1 index | | |  | The Shannon diversity | | |
| --- | --- | --- | --- | --- | --- | --- | --- |
|  | Beta coefficient | Standard error | *P* value |  | Beta coefficient | Standard error | *P* value |
| Random effects | | | | | | | |
| Age group | 0.22 | 0.02 | <0.001 |  | 0.05 | 0.05 | 0.19 |
| Fixed effects (ref.: 24 month-old group) | | | | | | | |
| 6 month-old group | -0.29 | 0.07 | <0.01 |  | -0.97 | 0.12 | <0.001 |
| 12 month-old group | -0.11 | 0.08 | 0.17 |  | -0.74 | 0.12 | <0.001 |

Notes: Random intercept and slope model was used with twins’ zygosity, sex, birth weight, gestational age, feeding pattern at 1 month-old, and maternal delivery age, educational level controlled for; Chao1 index was log2 transformed as non-normal distribution data.

Supplementary table 4. Microbiota with contribution rate for difference > 0.001 discovered by Simper analysis.

| Microbiota | 6 month-old group | | | | 12 month-old group | | | | 24 month-old group | | | |
| --- | --- | --- | --- | --- | --- | --- | --- | --- | --- | --- | --- | --- |
|  | means | SD | min | max | means | SD | min | max | means | SD | min | max |
| Enterococcus | 0.027763 | 0.037684 | 2.78E-05 | 0.261376 | 0.014418 | 0.061562 | 7.98E-05 | 0.977314 | 0.00601 | 0.008641 | 3.87E-05 | 0.061061 |
| Akkermansia | 0.018143 | 0.082433 | 0 | 0.829495 | 0.005116 | 0.032032 | 0 | 0.450745 | 0.003977 | 0.011868 | 0 | 0.115479 |
| Bifidobacterium | 0.292414 | 0.164057 | 0.007788 | 0.840287 | 0.264964 | 0.183553 | 0.001942 | 0.773856 | 0.130712 | 0.104157 | 0.005771 | 0.63042 |
| Escherichia_Shigella | 0.235309 | 0.147445 | 8.34E-05 | 0.736343 | 0.142305 | 0.124316 | 0.000266 | 0.651037 | 0.097268 | 0.104372 | 0.007863 | 0.674265 |
| Bacteroides | 0.056699 | 0.102731 | 0 | 0.616183 | 0.129457 | 0.171559 | 7.98E-05 | 0.706463 | 0.241996 | 0.171618 | 0.000794 | 0.664427 |
| Brevibacillus | 0.014619 | 0.043085 | 0 | 0.42579 | 0.012556 | 0.07307 | 0 | 0.687181 | 0.010065 | 0.044216 | 0 | 0.423475 |
| Megasphaera | 0.003492 | 0.023975 | 0 | 0.340231 | 0.006637 | 0.034469 | 0 | 0.415505 | 0.009374 | 0.058449 | 0 | 0.618336 |
| Parabacteroides | 0.009616 | 0.058044 | 0 | 0.622107 | 0.009875 | 0.043688 | 0 | 0.513325 | 0.014732 | 0.032947 | 0 | 0.266127 |
| Streptococcus | 0.02038 | 0.047054 | 0.000362 | 0.49605 | 0.037572 | 0.068062 | 0.000186 | 0.588617 | 0.01726 | 0.032594 | 0.000271 | 0.262486 |
| Prevotella | 0.001711 | 0.006242 | 0 | 0.0709 | 0.002368 | 0.008661 | 0 | 0.091755 | 0.012862 | 0.056996 | 0 | 0.513488 |
| Veillonella | 0.049489 | 0.071098 | 8.34E-05 | 0.552181 | 0.061741 | 0.084561 | 0.000133 | 0.496702 | 0.030748 | 0.043259 | 0.001085 | 0.289657 |
| Herbaspirillum | 0.031093 | 0.076182 | 0 | 0.444176 | 0.022634 | 0.101293 | 0 | 0.854761 | 0.042199 | 0.11008 | 0 | 0.844627 |
| Klebsiella | 0.035581 | 0.075816 | 0.000195 | 0.586894 | 0.011846 | 0.023315 | 0 | 0.18492 | 0.011483 | 0.017428 | 5.81E-05 | 0.11331 |
| Clostridium_sensu_stricto_1 | 0.012844 | 0.020197 | 0 | 0.154539 | 0.014803 | 0.035515 | 7.98E-05 | 0.382899 | 0.011414 | 0.013269 | 0.000174 | 0.088619 |
| Lactobacillus | 0.010488 | 0.035985 | 0 | 0.394749 | 0.006109 | 0.019017 | 0 | 0.207553 | 0.002586 | 0.005193 | 0 | 0.043651 |
| Ruminococcus_gnavus_group | 0.014681 | 0.025055 | 0 | 0.236816 | 0.036006 | 0.058284 | 0.000239 | 0.496702 | 0.015767 | 0.015524 | 0.00031 | 0.1234 |
| Faecalibacterium | 0.01487 | 0.03621 | 0 | 0.505062 | 0.0343 | 0.060909 | 0 | 0.580399 | 0.0911 | 0.08439 | 0.00093 | 0.47863 |
| Romboutsia | 0.003129 | 0.020698 | 0 | 0.345655 | 0.004491 | 0.009691 | 0 | 0.07867 | 0.004274 | 0.006896 | 3.87E-05 | 0.057304 |
| Haemophilus | 0.005873 | 0.017767 | 0 | 0.206275 | 0.009287 | 0.027214 | 0 | 0.318564 | 0.00757 | 0.01552 | 5.81E-05 | 0.14203 |
| CAG_352 | 0.000546 | 0.001635 | 0 | 0.018831 | 0.002546 | 0.020526 | 0 | 0.316489 | 0.003776 | 0.007067 | 0 | 0.045084 |
| Clostridioides | 0.007133 | 0.024551 | 0 | 0.30944 | 0.003356 | 0.008631 | 0 | 0.119122 | 0.0025 | 0.003538 | 0 | 0.021399 |
| Blautia | 0.009651 | 0.019147 | 0 | 0.229946 | 0.025911 | 0.045691 | 2.66E-05 | 0.312261 | 0.030138 | 0.04296 | 0.001123 | 0.297713 |
| Agathobacter | 0.001188 | 0.00236 | 0 | 0.014853 | 0.002652 | 0.010148 | 0 | 0.142287 | 0.013013 | 0.037583 | 0 | 0.307241 |
| Fusicatenibacter | 0.001644 | 0.004993 | 0 | 0.065421 | 0.005592 | 0.018071 | 0 | 0.170984 | 0.005987 | 0.02275 | 0 | 0.272595 |
| Ruminococcus | 0.00049 | 0.001227 | 0 | 0.01082 | 0.002201 | 0.016405 | 0 | 0.261516 | 0.003063 | 0.005949 | 0 | 0.04642 |
| Weissella | 6.52E-05 | 0.000439 | 0 | 0.004784 | 0.000992 | 0.015521 | 0 | 0.254096 | 1.87E-05 | 0.000132 | 0 | 0.001569 |
| Raoultella | 0.006927 | 0.036104 | 0 | 0.477025 | 0.001541 | 0.003772 | 0 | 0.035372 | 0.001809 | 0.005868 | 0 | 0.053237 |
| Lachnoclostridium | 0.003864 | 0.019453 | 0 | 0.218986 | 0.004041 | 0.008522 | 0 | 0.062447 | 0.004854 | 0.00729 | 3.87E-05 | 0.055755 |
| Dysgonomonas | 0.003122 | 0.018205 | 0 | 0.210698 | 0.001492 | 0.009976 | 0 | 0.115 | 0.001697 | 0.008276 | 0 | 0.083196 |
| Pseudomonas | 0.00074 | 0.010113 | 0 | 0.173509 | 0.00019 | 0.001714 | 0 | 0.026968 | 0.000202 | 0.000776 | 0 | 0.007669 |
| Kosakonia | 0.00092 | 0.002286 | 0 | 0.021223 | 0.00035 | 0.000886 | 0 | 0.007633 | 0.0016 | 0.014236 | 0 | 0.172396 |
| Erysipelatoclostridium | 0.005044 | 0.014196 | 0 | 0.140799 | 0.009253 | 0.017131 | 0.000133 | 0.166888 | 0.004092 | 0.006305 | 3.87E-05 | 0.051359 |
| Megamonas | 0.001277 | 0.00716 | 0 | 0.115988 | 0.00259 | 0.015314 | 0 | 0.159601 | 0.011653 | 0.054243 | 0 | 0.513353 |
| Enterobacter | 0.010936 | 0.034008 | 2.78E-05 | 0.460614 | 0.002706 | 0.004632 | 0 | 0.033777 | 0.003122 | 0.005169 | 5.81E-05 | 0.035595 |
| Collinsella | 0.002489 | 0.009373 | 0 | 0.074238 | 0.005644 | 0.020052 | 0 | 0.188085 | 0.0026 | 0.004929 | 0 | 0.023452 |
| Fusobacterium | 0.000231 | 0.000648 | 0 | 0.007037 | 0.000792 | 0.004934 | 0 | 0.07 | 0.002281 | 0.012635 | 0 | 0.124988 |
| Eubacterium_eligens_group | 0.000986 | 0.002633 | 0 | 0.027398 | 0.003825 | 0.014092 | 0 | 0.149388 | 0.005709 | 0.010684 | 0 | 0.083099 |
| Lachnospira | 0.000526 | 0.001464 | 0 | 0.016188 | 0.001457 | 0.007635 | 0 | 0.115559 | 0.002702 | 0.006545 | 0 | 0.045258 |
| Citrobacter | 0.007822 | 0.018389 | 2.78E-05 | 0.164191 | 0.003096 | 0.007597 | 0 | 0.074787 | 0.003263 | 0.008384 | 3.87E-05 | 0.064431 |
| Eubacterium_hallii_group | 0.000685 | 0.001588 | 0 | 0.017384 | 0.001954 | 0.006511 | 0 | 0.068112 | 0.003315 | 0.009397 | 0 | 0.103317 |
| Clostridium_innocuum_group | 0.003233 | 0.008332 | 0 | 0.103165 | 0.005636 | 0.010466 | 0 | 0.07633 | 0.001625 | 0.002274 | 0 | 0.014331 |
| Roseburia | 0.001351 | 0.00311 | 0 | 0.032988 | 0.002454 | 0.008813 | 0 | 0.116676 | 0.01026 | 0.027465 | 1.94E-05 | 0.245812 |
| Dialister | 0.000971 | 0.002439 | 0 | 0.024755 | 0.001328 | 0.00307 | 0 | 0.021356 | 0.01163 | 0.026099 | 0 | 0.190522 |
| Subdoligranulum | 0.001707 | 0.007253 | 0 | 0.110842 | 0.002325 | 0.008407 | 0 | 0.078192 | 0.008898 | 0.015737 | 0 | 0.088096 |
| TM7x | 0.000439 | 0.003428 | 0 | 0.057187 | 0.000515 | 0.004863 | 0 | 0.076516 | 0.000267 | 0.001834 | 0 | 0.021787 |
| Alloprevotella | 0.000108 | 0.000319 | 0 | 0.002392 | 0.00012 | 0.000348 | 0 | 0.003245 | 0.000675 | 0.006141 | 0 | 0.074152 |
| Intestinibacter | 0.001971 | 0.004645 | 0 | 0.071178 | 0.002808 | 0.006299 | 0 | 0.06891 | 0.002533 | 0.002273 | 5.81E-05 | 0.01375 |
| Clostridium_sensu_stricto_2 | 0.000512 | 0.004729 | 0 | 0.064809 | 6.41E-05 | 0.000267 | 0 | 0.002261 | 5.34E-05 | 0.000237 | 0 | 0.002188 |
| Sphingomonas | 0.001205 | 0.004325 | 0 | 0.063195 | 0.000786 | 0.002385 | 0 | 0.022926 | 0.000829 | 0.001642 | 0 | 0.012917 |
| Anaerostipes | 0.001269 | 0.00429 | 0 | 0.068564 | 0.003497 | 0.006623 | 0 | 0.059309 | 0.005132 | 0.007457 | 7.75E-05 | 0.060635 |
| Sellimonas | 0.00118 | 0.004365 | 0 | 0.060553 | 0.001112 | 0.002903 | 0 | 0.034628 | 0.001763 | 0.003164 | 0 | 0.021942 |
| unidentified_Ruminococcaceae | 0.000792 | 0.00404 | 0 | 0.060219 | 0.000318 | 0.001901 | 0 | 0.027872 | 0.000351 | 0.000798 | 0 | 0.006546 |
| Flavonifractor | 0.002809 | 0.005813 | 0 | 0.057549 | 0.005002 | 0.011703 | 0 | 0.145372 | 0.004759 | 0.004368 | 0.000116 | 0.021825 |
| Aeromonas | 0.000216 | 0.003251 | 0 | 0.055852 | 2.59E-05 | 0.000122 | 0 | 0.001702 | 5.39E-05 | 0.000381 | 0 | 0.004396 |
| UBA1819 | 0.001269 | 0.004683 | 0 | 0.053432 | 0.001736 | 0.005654 | 0 | 0.059814 | 0.001917 | 0.003393 | 0 | 0.021225 |
| Phascolarctobacterium | 0.000421 | 0.003291 | 0 | 0.053293 | 0.000707 | 0.00346 | 0 | 0.048085 | 0.001637 | 0.00773 | 0 | 0.083216 |
| Parasutterella | 0.000927 | 0.002131 | 0 | 0.017662 | 0.005853 | 0.035994 | 0 | 0.465532 | 0.005981 | 0.009707 | 0 | 0.062262 |
| Desulfovibrio | 0.000237 | 0.002678 | 0 | 0.045394 | 0.000125 | 0.000565 | 0 | 0.004521 | 0.000377 | 0.001352 | 0 | 0.011097 |
| Hungatella | 0.000319 | 0.002813 | 0 | 0.043447 | 0.000284 | 0.001414 | 0 | 0.019548 | 4.06E-05 | 9.05E-05 | 0 | 0.000542 |
| Prevotellaceae_NK3B31_group | 0.000113 | 0.00137 | 0 | 0.022864 | 0.000194 | 0.002564 | 0 | 0.041755 | 0.000231 | 0.001206 | 0 | 0.00978 |
| Candidatus_Competibacter | 0.000236 | 0.002777 | 0 | 0.038663 | 0.000224 | 0.002518 | 0 | 0.033085 | . | . | . |  |

Supplementary table 5. Correlation between the distinct gut microbiota groups and the distinct BMI_Z group

| Distinct microbiota groups | r | *p* |
| --- | --- | --- |
| Anaerostipes | 0.15408 | **0.0278** |
| Parabacteroides | -0.15047 | **0.0317** |
| Akkermansia | -0.13858 | **0.0481** |
| Clostridium_innocuum_group | 0.13759 | **0.0497** |
| Roseburia | 0.13759 | **0.0497** |
| Brevibacillus | 0.12997 | 0.0639 |
| Streptococcus | 0.12933 | 0.0652 |
| Subdoligranulum | 0.11958 | 0.0885 |
| Eubacterium_eligens_group | 0.11119 | 0.1133 |
| Eubacterium_hallii_group | -0.11119 | 0.1133 |
| Sphingomonas | 0.10344 | 0.1409 |
| Lactobacillus | 0.09425 | 0.18 |
| Enterobacter | -0.08325 | 0.2365 |
| Haemophilus | -0.08132 | 0.2476 |
| Veillonella | -0.07844 | 0.2648 |
| CAG_352 | -0.07104 | 0.3126 |
| Enterococcus | 0.06633 | 0.3459 |
| Faecalibacterium | -0.06595 | 0.3487 |
| Romboutsia | -0.06595 | 0.3487 |
| Prevotella | 0.06335 | 0.368 |
| Herbaspirillum | -0.05682 | 0.4195 |
| Klebsiella | -0.05682 | 0.4195 |
| Erysipelatoclostridium | -0.04888 | 0.4875 |
| Agathobacter | 0.04121 | 0.5584 |
| Fusicatenibacter | -0.03278 | 0.6416 |
| Bacteroides | -0.03097 | 0.6602 |
| Collinsella | 0.02202 | 0.7546 |
| Raoultella | -0.01733 | 0.8057 |
| Dialister | 0.00417 | 0.9528 |
| Clostridioides | -0.00374 | 0.9576 |

Notes: r-Spearman correlation index.

Supplementary table 6. Association between distinct trajectory curve groups of gut microbiota and BMI_Z: results from generalized estimation equation models.

|  | Un-adjusted model | | | | Adjusted model | | | |
| --- | --- | --- | --- | --- | --- | --- | --- | --- |
|  | Co-efficient | 95% CI | *P* | FDR-*P*^*^ | Co-efficient | 95% CI | *P* | FDR-*p* |
| *Akkermansia* | 0.89 | -0.01, 1.80 | 0.05 | 0.05 | 0.85 | -0.17, 1.87 | 0.10 | 0.125 |
| *Parabacteroides* | 0.70 | 0.01, 1.39 | **0.04** | 0.05 | 0.75 | 0.02, 1.49 | **0.04** | 0.125 |
| *Clostridium innocuum group* | -0.57 | -1.15, 0.01 | 0.05 | 0.05 | -0.57 | -1.21, 0.06 | 0.07 | 0.125 |
| *Roseburia* | 0.57 | -0.01, 1.15 | 0.05 | 0.05 | 0.57 | -0.06, 1.21 | 0.08 | 0.125 |
| *Anaerostipes* | -0.64 | -1.22, -0.07 | **0.03** | 0.05 | -0.47 | -1.11, 0.17 | 0.15 | 0.125 |

Note: In the adjusted models, twins’ zygosity, sex, birth weight, gestational age, feeding pattern at 1 month old, maternal delivery age and educational level were controlled for;

*: false discovery rate.

Supplementary Figure 1. Heatmap of the 35 most common bacterial among the three age groups at the phylum and genus levels.

M6: 6 months old group; M12: 12 months old group; M24: 24 months old group.

A refers to genus level, B refers to phylum level.

Appendix1: introductions about model selection in group-based trajectory models (GBTM).

GBTM were modulated in SAS 9.4 with TRAJ package. We firstly identified the optimal number of trajectory groups using the Bayesian information criteria (BIC). We fitted models with different numbers, from one to four, of trajectory groups using a quadratic form for all trajectories, and generated the corresponding BICs. Then we used the BICs to determine the optimal number of groups, the higher BIC indicates better model fitting and a difference lower than 2 means not enough evidence against the null model and was used as the optimal group number. Then linear, quadratic, cubic, and quartic functions were used to best describe the observed trajectories. Because we had only three measurements, the cubic and quartic functions were not enough to capture the variations in trajectories. Compared with the linear function, the quadratic function was better fitted. At last, the quadratic function was finally selected to describe the trajectory shape. After obtaining the optimal number of the trajectory groups and the proper trajectory shape, we selected the group that had a stable trend far from the null score as high DTC group and these with a stable trend near the null score as low DTC group for both log transformed microbiota genera and BMI_Z.

Appendix2: computer scripts for group-based trajectory models and generalized estimation equation models in SAS 9.4.

title ‘Building the distinct trajectory curves of BMI_Z’;

**PROC** **TRAJ** DATA=microbiota OUTPLOT=OP OUTSTAT=OS OUT=OF OUTEST=OE ITDETAIL

ID cohort_ID; VAR g1-g3; INDEP month1-month3;

MODEL cnorm; min -**100**; MAX **100**; NGROUPS **2**; ORDER **2** **2** ;

**run**;

title 'Building the association models for distinct trajectory curves of log2 transformed microbiota genera and BMI_Z';

**proc** **genmod** data=microbiota_distinct;

class cohort_ID GROUP_Parabacteroides feeding1 delivery_mode maternal_Education zygosity;

model GROUP_bmiz =GROUP_Parabacteroides sex Weight0 feeding1 delivery_mode gest_age agem maternal_Education zygosity /dist=binomial link=logit type3;

repeated subject=cohort_ID /type=ind modelse;

**run**;
